# Molecular insights into the compatible and incompatible interactions between sugar beet and the beet cyst nematode

**DOI:** 10.1186/s12870-020-02706-8

**Published:** 2020-10-22

**Authors:** Razieh Ghaemi, Ebrahim Pourjam, Naser Safaie, Bruno Verstraeten, Seyed Bagher Mahmoudi, Rahim Mehrabi, Tim De Meyer, Tina Kyndt

**Affiliations:** 1 Department of Plant Pathology, Faculty of Agriculture, Tarbiat Modares University, Tehran, Iran; 2 Department of Biotechnology, Ghent University, Coupure Links 653, B-9000, Ghent, Belgium; 3 Sugar Beet Seed Institute, Agricultural Research, Education and Extension Organization (AREEO), Karaj, Iran; 4 Department of Biotechnology, College of Agriculture, Isfahan University of Technology, P.O. Box 8415683111, Isfahan, Iran; 5 Department of Data Analysis and Mathematical Modelling, Ghent University, Coupure Links 653, B-9000 Ghent, Belgium

**Keywords:** Sugar beet, *Heterodera schachtii*, CYSTM domain-containing proteins, Ethylene, Jasmonate, Transcriptome

## Abstract

**Background:**

Sugar beet (*Beta vulgaris* subsp. *vulgaris*) is an economically important crop that provides nearly one third of the global sugar production. The beet cyst nematode (BCN), *Heterodera schachtii*, causes major yield losses in sugar beet and other crops worldwide. The most effective and economic approach to control this nematode is growing tolerant or resistant cultivars. To identify candidate genes involved in susceptibility and resistance, the transcriptome of sugar beet and BCN in compatible and incompatible interactions at two time points was studied using mRNA-seq.

**Results:**

In the susceptible cultivar, most defense-related genes were induced at 4 dai while suppressed at 10 dai but in the resistant cultivar Nemakill, induction of genes involved in the plant defense response was observed at both time points. In the compatible interaction, alterations in phytohormone-related genes were detected. The effect of exogenous application of Methyl Jasmonate and ET-generator ethephon on susceptible plants was therefore investigated and the results revealed significant reduction in plant susceptibility. Genes putatively involved in the resistance of Nemakill were identified, such as genes involved in phenylpropanoid pathway and genes encoding CYSTM domain-containing proteins, F-box proteins, chitinase, galactono-1,4-lactone dehydrogenase and CASP-like protein. Also, the transcriptome of the BCN was analyzed in infected root samples and several novel potential nematode effector genes were found.

**Conclusions:**

Our data provides detailed insights into the plant and nematode transcriptional changes occurring during compatible and incompatible interactions between sugar beet and BCN. Many important genes playing potential roles in susceptibility or resistance of sugar beet against BCN, as well as some BCN effectors with a potential role as *avr* proteins were identified. In addition, our findings indicate the effective role of jasmonate and ethylene in enhancing sugar beet defense response against BCN. This research provides new molecular insights into the plant-nematode interactions that can be used to design novel management strategies against BCN.

**Supplementary information:**

**Supplementary information** accompanies this paper at 10.1186/s12870-020-02706-8.

## Background

Sugar beet (*Beta vulgaris subsp. vulgaris*) is a biennial, outbreeding and diploid (2n = 18) plant from the family Amaranthaceae, that is cultivated in temperate and subtropical regions [1]. Sugar beet is one of the most important crops worldwide, grown in 58 countries [2], and providing about 30% of the total world sugar production. It is also important as a source for bioethanol and animal feed [1].

Many crops are damaged by different diseases and pests including plant-parasitic nematodes. The annual global crop losses caused by plant-parasitic nematodes have been estimated at 157 billion dollars [3]. The beet cyst nematode (BCN, *Heterodera schachtii* [4]) has been identified as a plant pathogen since 1859 in Germany [5] and is now widely distributed throughout most of the beet-growing areas in the world, causing considerable yield losses (up to 60%). Infected beet plants exhibit symptoms including stunting and reduced growth, wilted leaves, and abnormal root development, also known as “bearded roots” [6]. The host range of the BCN is very wide as it can infect more than 200 plant species, mainly plants of the families Amaranthaceae (many species of *Beta* and *Chenopodium*) and Brassicaceae (e.g. *Brassica oleracea*, *B. napus*, *B. rapa*, *Rhaphanus sativus* and *Arabidopsis* sp.) [7].

The second-stage juvenile (J2) of the BCN penetrates the host root and migrates intracellularly through the cortical cells towards the vascular cylinder to find a proper cell for feeding site induction. After selection of a single cell as initial syncytial cell (ISC), it secretes molecules through its stylet and starts feeding from the ISC. Then, partial cell wall dissolution and protoplast fusion of several hundred neighboring cells occurs, leading to the formation of a highly metabolic active and multinucleate syncytium. The syncytiaum is the only nutrition source throughout the nematode‘s life. After starting the feeding process, the J2 becomes sedentary and matures after three molts [8]. Adult males leave their syncytia in the roots to mate with females while female nematodes remain attached to the feeding site and, following fertilization, produce several hundred eggs inside their enlarged body. After the completion of egg development, females die and their body wall hardens to form a cyst, which protects the eggs until hatching [9]. Completing the life cycle depends on the successful induction and maintenance of the feeding structure. Nematode secretions from esophageal glands, amphids and cuticle cause cellular reprogramming events related to major changes in the plant gene expression profile. Several nematode effectors from root-knot and cyst nematodes, such as cellulases, pectinases, expansins, chorismate mutase and calreticulin, have been reported [10–15].

Phytohormones play important roles in the formation of nematode feeding sites and regulation of gene expression in plant defense/susceptibility responses [16–20]. The role of salicylic acid (SA) in plant defense against plant-parasitic nematodes has been investigated in Arabidopsis. Wubben et al. [19] found that SA-deficient mutants of Arabidopsis exhibited increased susceptibility to BCN and SA-treated wild type plants showed decreased BCN infection. Kammerhofer et al. [17] suggested that SA does not play a major role early during *H. schachtii* infection, but can suppress syncytium and female development at later time points. Moreover, Kammerhofer et al. [17] showed that mainly jasmonic acid (JA) can trigger plant defense against BCN in Arabidopsis. Also in other plant species, a number of studies have shown that exogenous application of JA on roots or shoots of plants enhanced resistance to plant parasitic nematodes [21–23]. In Arabidopsis, Ethylene (ET) positively affects BCN attraction to the root [17] and a positive role for ET in syncytium formation has also been demonstrated [20, 24].

The transcriptional changes occurring during the compatible Arabidopsis-*H. schachtii* interaction have been studied using differential display and microarrays [25–27]. Puthoff et al. [26] used Affymetrix GeneChip microarrays to compare gene expression in whole roots of Arabidopsis infected by *H. schachtii* or *Heterodera glycines* (soybean cyst nematode, SCN) at 3 days after infection (dai) and identified 128 and 12 genes, respectively, with altered mRNA levels following the BCN or SCN infection. Szakasits et al. [27] reported that gene expression in syncytia induced by *H. schachtii* in Arabidopsis roots did not strongly differ when comparing two time points, 5 and 15 dai. They analyzed the expression of 21,138 genes and identified 3893 and 3338 genes, respectively, with higher or lower expression levels in syncytia compared with control roots. Their results revealed that genes involved in degradation of cell walls (such as pectate lyases and expansins), chloroplast proteins (such as glyceraldehyde 3-phosphate dehydrogenase A, cytochrome B6-F complex iron-sulfur subunit) and chlorophyll a-b binding proteins were up-regulated. Among the strongly down-regulated genes, peroxidases and major intrinsic proteins (including aquaporins) were observed.

Although sugar beet is the main host of *H. schachtii*, there is limited information about the alterations of gene expression in this host plant, either in susceptible or resistant plants. Resistance to BCN has not been found in cultivated beets. However, some commercial resistant varieties (eg. Nemakill, Evasion, Nematop) have been generated through interspecific crosses between *B. vulgaris* and the wild relative species *Patellifolia procumbens* [28–31]. The resistant varieties contain a translocated fragment of *P. procumbens* chromosome 1 harboring the resistance gene *Hs1*^*pro-1*^. Although *Hs1*^*pro-1*^ is the first cloned nematode resistance gene and encodes a 282–amino acid protein with leucine-rich repeats (LRR) and a transmembrane domain, its role in the resistance of sugar beet against BCN is still in doubt [32]. The existence of a second nematode resistance gene named *Hs1–2* in the vicinity of *Hs1*^*pro-1*^ has been suggested although further characterization is lacking [33, 34].

Resistant sugar beets are invaded by J2 of *H. schachtii* and can establish a feeding site, but syncytia degenerate before nematode maturation, hence hindering nematodes to complete their lifecycle [32, 35]. Samuelian et al. [36] used the cDNA-AFLP technique to identify sugar beet genes induced upon infection with the BCN. They analyzed 8000 transcript-derived fragments (TDFs) from infected hairy root clones of susceptible and resistant sugar beet (carrying the resistance gene *Hs1*^*pro-1*^). They found that TDF_6, *Beta vulgaris Ki1*, was differentially expressed in both materials but more strongly in resistant plants. Upon transgenic over-expression this gene was able to inhibit the development of BCN in susceptible hairy roots.

Considering the importance of sugar beet and *H. schachtii* as a major limiting factor of beet production, our research aimed at performing a transcriptome study of compatible and incompatible interactions of sugar beet with BCN at two time points. The results revealed expression changes in genes involved in defense response, hormone pathways, metabolism, nutrition, and transcription regulation. Also, our experiments showed that exogenous application of methyl jasmonate and ET-generator ethephon on the susceptible sugar beet plants causes induced defence against BCN. Next to that, we have identified putative candidate genes that are involved in the resistance of cultivar Nemakill and nematode effectors that are expressed during infection in either a susceptible or a resistant cultivar.

## Results

### Sequencing and mapping

In this study, mRNA of root tissues of uninfected and BCN-infected susceptible and resistant sugar beet plants was sequenced at two time points upon inoculation: 4 days after inoculation (dai, early stage) and 10 dai (late stage), and in 2 independent biological replicates leading to a total of 16 sequenced samples. In a compatible interaction, the nematodes are at the J2 stage at 4 dai, while at 10 dai they are at the late J3 stage. As susceptible cultivar we worked with line 7112*SB36 (Sugar Beet Seed Institute, Iran), while the resistant cultivar was Nemakill [29, 31, 37, 38].

**Fig. 1:**
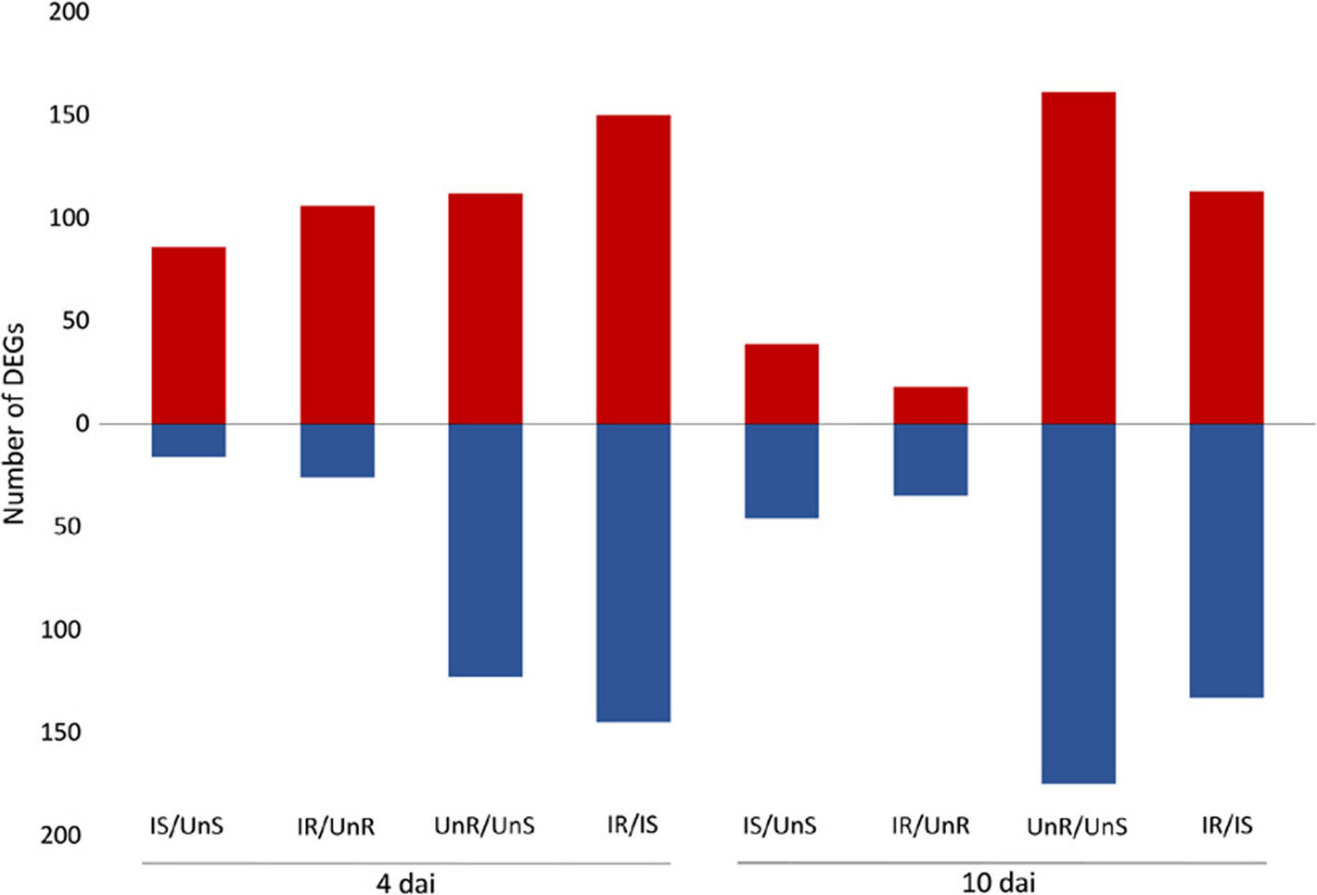
Number of significant differentially expressed genes (DEGs) identified by all pairwise comparisons executed in this study, to analyze sugar beet in interaction with *Heterodera schachtii*. Red: up-regulated; blue: down-regulated genes. IS, infected susceptible roots; UnS, uninfected susceptible roots; IR, infected resistant roots; UnR, uninfected resistant roots; dai, days after inoculation

A total of 442,691,707 raw reads were obtained from the transcriptome sequencing. The average number of trimmed reads per sample was 26,581,145. A total of 425,298,335 trimmed reads that were 73–74 bp in length were aligned against the reference genome sequence of sugar beet (Refbeet1.1, [1]) using STAR and 90–91% of the sequenced reads could be uniquely mapped across all samples. The total number of uniquely mapped reads was 385,810,355, representing an average per sample coverage of 7.33X of the sugar beet transcriptome. An overview of the sequencing data and mapping results is shown in Table 1. The sequencing data have been deposited at the National Center for Biotechnology Information (NCBI) Gene Expression Omnibus (GEO) under the accession number (GSE135555). The number of differentially expressed genes (DEGs) obtained by all executed pairwise comparisons is shown in Fig. 1, and is described in detail below.

**Table 1:** Summary of the transcriptome data from sugar beet roots in the compatible and incompatible interaction with *Heterodera schachtii*

Sample	Number of trimmed reads	Number of uniquely mapped reads	Percentages of uniquely mapped reads
Uninfected susceptible roots at 4 dai (UnS-4 dai)	55,453,474	50,263,420	90.66
Infected susceptible roots at 4 dai (IS-4 dai)	54,891,104	49,904,470	90.71
Uninfected resistant roots at 4 dai (UnR-4 dai)	52,935,813	48,014,740	90.74
Infected resistant roots at 4 dai (IR-4 dai)	50,732,663	46,245,798	91.19
Uninfected susceptible roots at 10 dai (UnS-10 dai)	55,008,558	50,229,976	91.29
Infected susceptible roots at 10 dai (IS-10 dai)	49,755,520	45,227,314	90.88
Uninfected resistant roots at 10 dai (UnR-10 dai)	54,885,472	49,741,102	90.63
Infected resistant roots at 10 dai (IR-10 dai)	51,635,731	46,183,535	90.25
Total	425,298,335	385,810,355	
Transcriptome size	246,730,718 bp		
Average per sample coverage of the sugar beet transcriptome		7.33X

### Differentially expressed genes (DEGs) in the compatible sugar beet-BCN interaction

#### Early stage (4 dai)

**Fig. 2:**
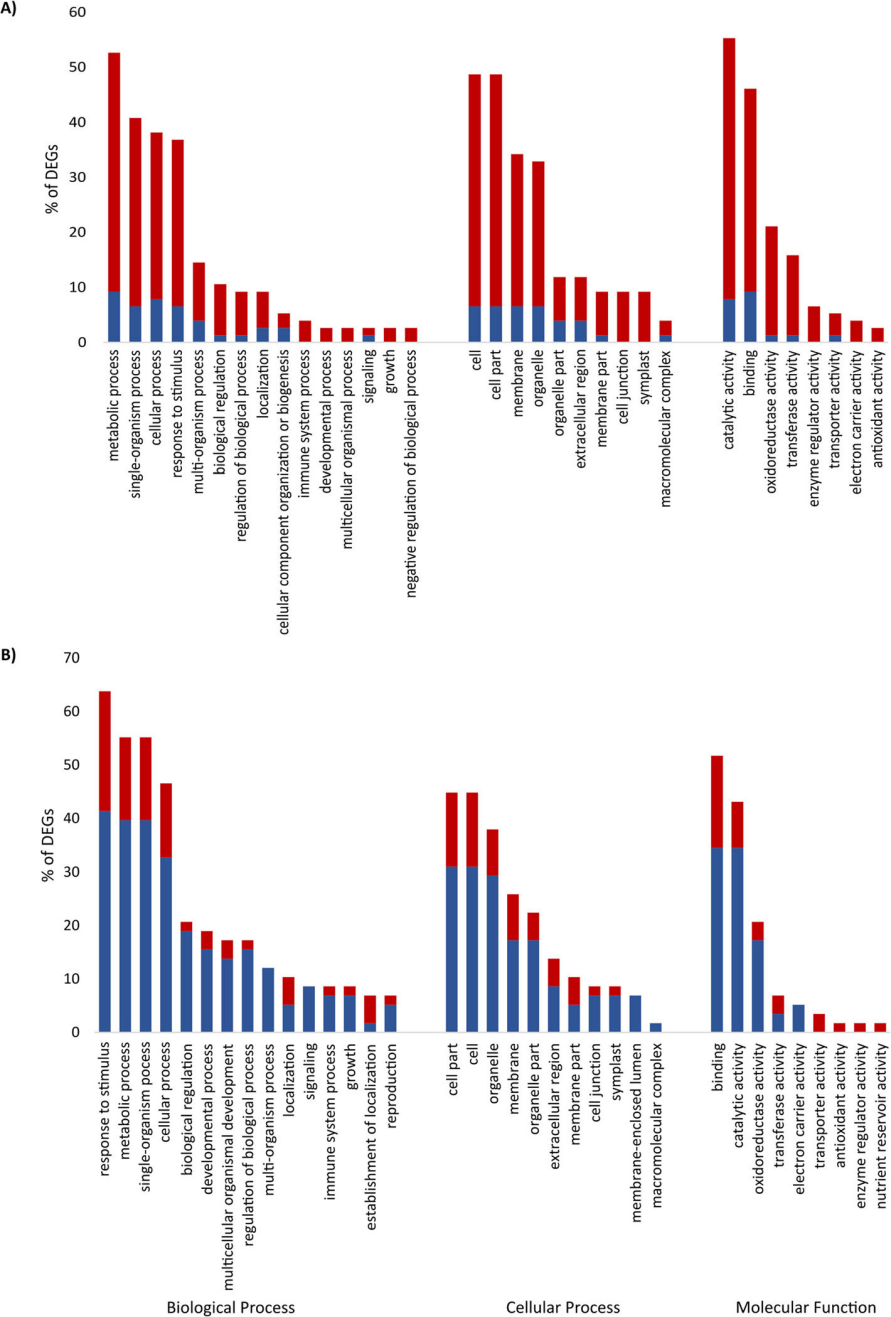
Classification of significant DEGs obtained from the BCN-infected susceptible sugar beet roots compared to the uninfected susceptible sugar beet roots at **a** 4 days after inoculation (IS vs UnS-4 dai) and **b** 10 days after inoculation (IS vs UnS-10 dai) in three main Gene Ontology (GO) categories. Up-regulated and down-regulated GO terms are shown in red and blue bars, respectively

One hundred two transcripts were significantly differentially expressed in the infected susceptible roots vs their corresponding uninfected controls at 4 dai, of which 86 genes were up-regulated and 16 down-regulated, indicating a general pattern of induction of genes at this early stage of BCN infection (Additional file 1: Table S1). It should be noted that among these 102 DEGs, 34 genes were not functionally annotated in PLAZA 3.0. Gene set analysis revealed particular enrichment for GO terms, metabolic process, single-organism process, cellular process, response to stimulus, multi-organism process, catalytic activity, binding, oxidoreductase activity and transferase activity (Fig. 2).

#### Late stage (10 dai)

At the later time point (10 dai), 85 DEGs were identified, of which 39 genes were up-regulated and 46 genes were down-regulated (Additional file 1: Table S1). The GO terms, response to stimulus, metabolic process, single-organism process, cellular process, biological regulation, binding, catalytic activity, oxidoreductase activity and transferase activity, were the most enriched (Fig. 2).

#### General changes in the compatible interaction

Comparing the transcriptome of infected roots vs uninfected controls at both time points in the compatible interaction showed significant alterations in the expression of genes involved in metabolism, cell wall architecture, developmental process, transport, plant defense responses, transcription factors and hormone metabolism. Genes related to cell wall architecture such as pectinesterase and polygalacturonase (cell wall degradation), glycine-rich proteins (cell wall proteins) and glycosyltransferases (cell wall synthesis) were significantly up-regulated at both time points (Additional file 2: Table S2). Also, expression of transporter genes including lipid transporters, ion transporters, ABC transporter, peptide and nitrate transporters was significantly changed at both time points (Additional file 2: Table S2). In addition, the expression of some genes involved in plant hormone metabolism, including genes related to auxin (e.g. indole acetic acid, IAA), gibberellic acid (GA), abscisic acid (ABA) and cytokinin (CK), as well as SA, JA and ET was altered (Additional file 2: Table S2). When focusing on genes involved in the plant defense response, we observed that the majority of these genes were induced at the early stage (4 dai), while suppressed at the later stage (10 dai) in the susceptible cultivar. For example, genes encoding NADPH-cytochrome P450 reductase and trans-cinnamate 4-monoxygenase - involved in the phenylpropanoid pathway were up and down-regulated at 4 and 10 dai, respectively (Additional file 2: Table S2). The expression of four proteinase inhibitor genes belonging to the Kunitz family trypsin and protease inhibitor protein family was up-regulated at 4 dai. Genes encoding Defensin-like proteins, glutathione S-transferase genes (GST), two BRASSINOSTEROID INSENSITIVE 1-associated receptor (BAK1) genes and some peroxidase genes were induced at both stages. On the other hand, genes encoding chitinase and galacto-1,4-lactone dehydrogenase were down-regulated at 4 dai and two amorpha-4,11-diene-12 monoxygenase genes, several heat shock proteins (HSP), two DIBOA-glucoside dioxygenase BX6 genes - involved in benzoxazinone synthesis - were suppressed at 10 dai. The analysis also revealed alteration in the expression of some transcription factors (TFs) in the susceptible sugar beet plants: a gene encoding a zinc finger TF was up and down-regulated at 4 and 10 dai, respectively. The expression of two genes encoding basic helix-loop-helix family (bHLH) TFs were up-regulated at 4 dai.

Two linoleate 9S-lipoxygenase genes, involved in JA biosynthesis genes were up-regulated at 4 dai. Three ET-responsive genes such as one APETALA2/ethylene-responsive transcription factor (AP2/ERF) gene were down-regulated in the infected roots at 10 dai (Additional file 2: Table S2).

**Fig. 3:**
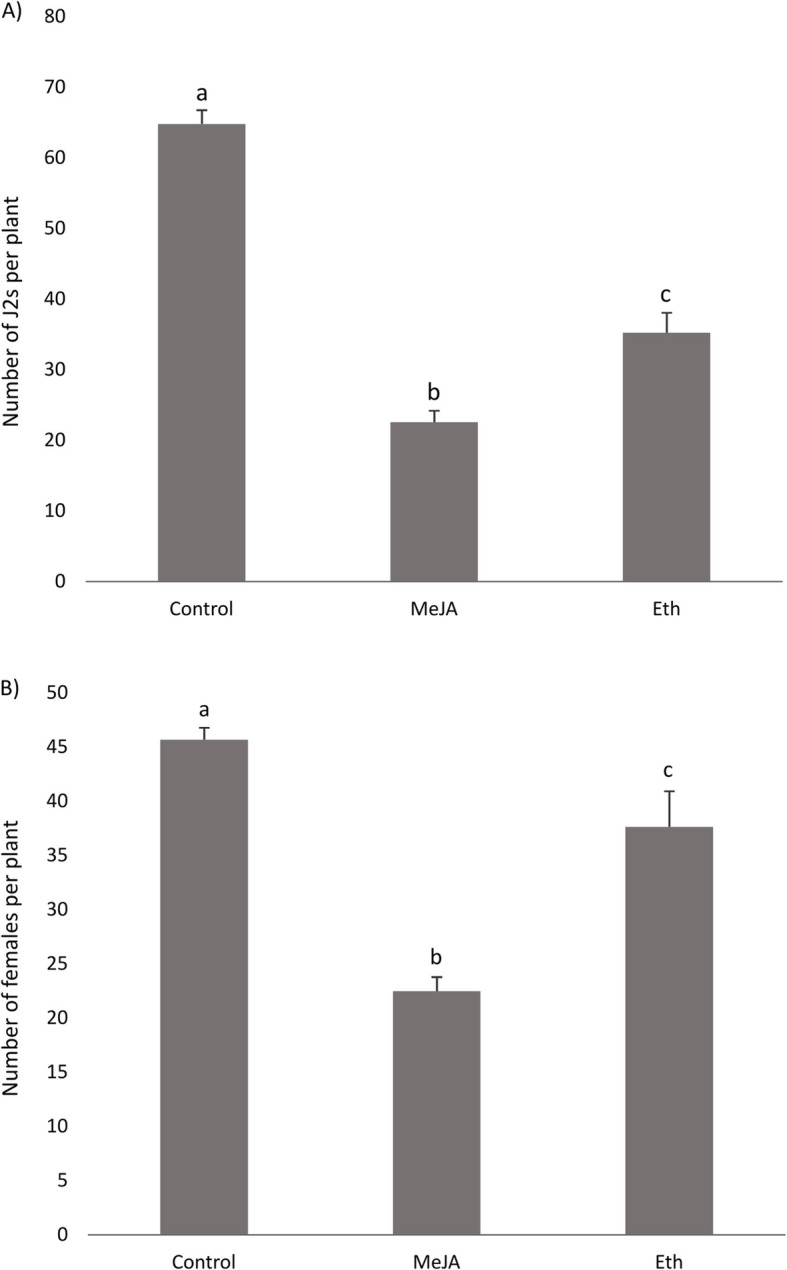
Effects of exogenous application of methyl jasmonate (MeJA, 100 μM) or ethephon (Eth, 500 μM) on invasion and development of *Heterodera schachtii* in susceptible sugar beet plants (line 7112*SB36). **a** infection rate at 4 days after inoculation (invasion) and **b** number of females at 21 days after inoculation (development). Chemicals were applied 24 h before nematode inoculation. Bars represent means + SE of 10 plants. Different letters indicate statistically significant differences (*P* < 0.05). The experiment was independently repeated three times, with similar results, except for the female number at 21 dai upon Eth treatment, which was not significantly affected in one of the repeats

Considering the alterations of some genes involved in plant defense hormones ET and JA in BCN infected roots and the lack of knowledge on the role of these hormones in sugar beet defense against BCN, the effect of external stimulation of these hormones on sugar beet immunity to BCN was investigated. To this aim, we treated the susceptible sugar beet with MeJA, or ET-generator Ethephon, 24 h prior to inoculation. The number of J2s and females at 4 and 21 dai, respectively, were compared on treated and control plants. Upon exogenous application of Eth, the number of J2s per plant (4 dai) was lower in Eth-treated plants compared to controls. However, the results obtained at the later time point (21 dai), where females were counted, were inconsistent between experiments (Fig. 3). On the other hand, the results showed that MeJA-treated plants had significantly fewer J2s (at 4 dai) and females (at 21 dai) per plant than control plants in all experiments.

### Differentially expressed genes (DEGs) in the incompatible sugar beet-BCN interaction in cultivar Nemakill

#### Early stage (4 dai)

**Fig. 4:**
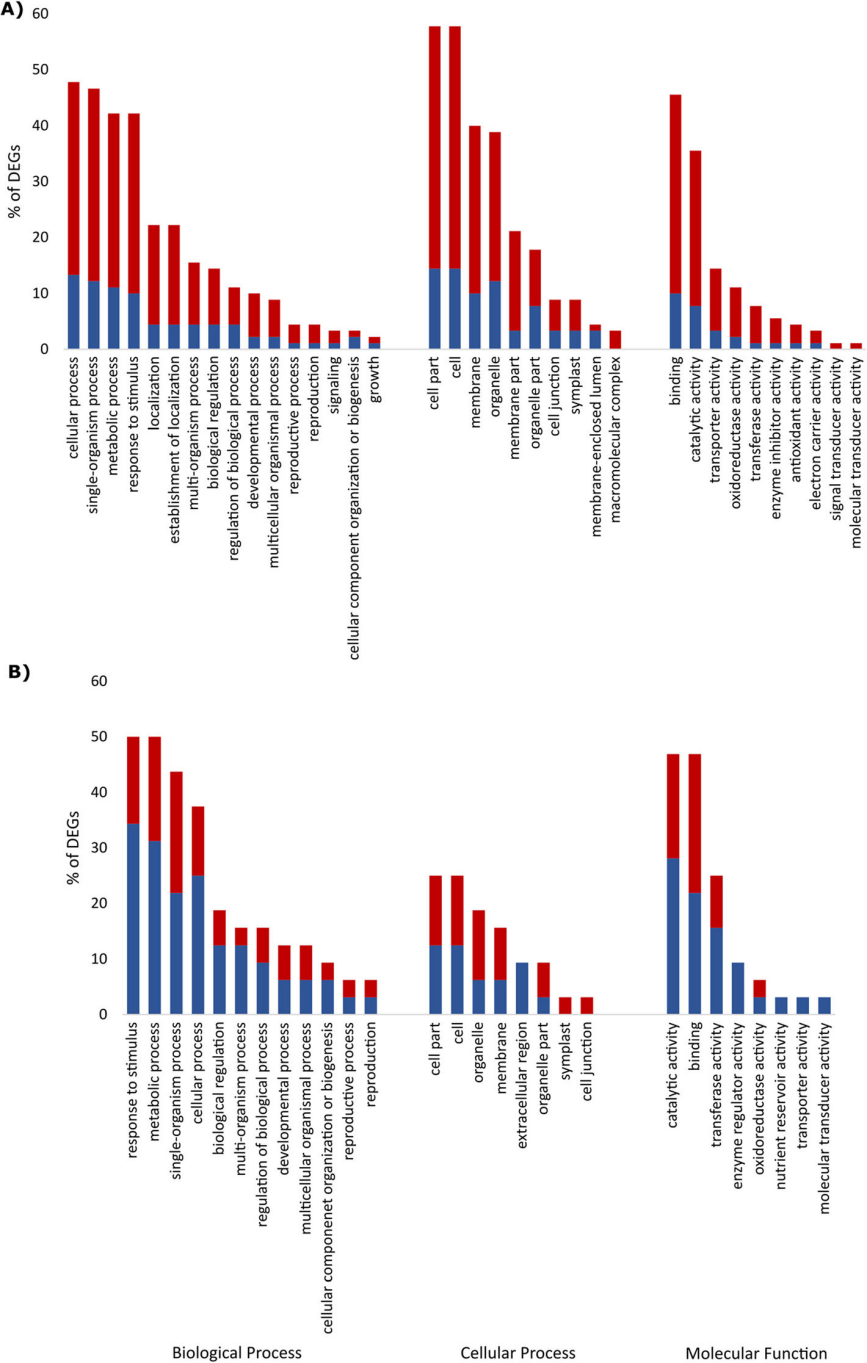
Classification of significant DEGs obtained from the BCN-infected resistant sugar beet roots compared to uninfected resistant sugar beet roots at **a** 4 days after inoculation (IR vs UnR-4 dai) and **b** 10 days after inoculation (IR vs UnR-10 dai) in three main Gene Ontology (GO) categories. Up-regulated and down-regulated GO terms are shown in red and blue bars, respectively

Comparing the transcriptome of infected roots of the resistant cultivar Nemakill with uninfected roots at 4 dai, 132 DEGs were identified, of which 106 genes were up and 26 genes were down-regulated (Additional file 3: Table S3), again showing a general pattern of gene induction. Forty eight of these genes were not functionally annotated in PLAZA 3.0. Gene set analysis revealed particular enrichment for following GO terms, cellular process, single-organism process, metabolic process, response to stimulus, localization, multi-organism process, binding, catalytic activity, transporter activity, oxidoreductase activity and transferase activity (Fig. 4).

#### Late stage (10 dai)

At 10 dai, 53 transcripts were differentially expressed, of which 18 genes were induced and 35 genes were suppressed (Additional file 3: Table S3). The GO terms, response to stimulus, metabolic process, single-organism process, cellular process, biological regulation, catalytic activity, binding, transferase activity and enzyme regulator activity, were the most enriched (Fig. 4).

#### General changes in the incompatible interaction

When considering both time points, the results generally showed changes in the expression of genes related to metabolism, cell wall architecture, transport, plant defense responses, transcription factors and hormone metabolism. The expression of some genes involved in cell wall architecture, including genes involved in cell wall degradation (polygalacturonase, xylan 1,4-beta-xylosidase, cell wall-associated hydrolase and expansin) and cell wall proteins (arabinogalactan peptide, glycine-rich proteins and proline-rich proteins) were altered (Additional file 4: Table S4). Several genes encoding transporters - amino acid transporter, sugar transporter, lipid transporters, peptide transporters, aquaporins, ABC transporters and ion transporters - were induced at 4 dai (Additional file 4: Table S4). When focusing on plant defense response genes, proteinase inhibitor genes of the kunitz family trypsin and protease inhibitor proteins, defensin-like protein, lignin-forming anionic peroxidases, endochitinase, dirigent protein 23, BURP domain-containing proteins, PR1, PR6, CYSTM domain-containing proteins and a ‘cell killing protein’ showed up-regulation in the incompatible interaction. Also, two genes of the zinc finger TF family, and a gene encoding basic-leucine zipper (bZIP) TF were induced in the infected sugar beet roots (Additional file 4: Table S4). The expression of three genes involved in IAA biosynthesis were up-regulated at 4 dai. Genes related to ABA (2 at 4 dai and 1 gene at 10 dai) were induced in the infected roots compared to the uninfected samples. Moreover, a JA-responsive gene was up-regulated at 4 dai while some JA-induced proteins were suppressed at 10 dai (Additional file 4: Table S4).

In addition, the expression of three genes containing a leucine-rich repeat (LRR) domain were up-regulated at 10 dai (Additional file 4: Table S4). The *Hs1*^*pro-1*^-gene was strongly expressed under both infected and uninfected conditions in the resistant cultivar.

### Comparison of the sugar beet transcriptome between compatible and incompatible interactions

Firstly, it should be mentioned that, since the genome of sugar beet has been sequenced only recently [1], there is no well-annotated genome available. Secondly, the sugar beet genotypes which were used in this study have a different genetic background making it challenging to compare their transcriptional response to BCN infection. Therefore, the transcriptome of two sugar beet cultivars was first compared in uninfected root systems at both time points, to elucidate general differences related to this varying genetic background. The results indicated that 236 genes were significantly differentially expressed between uninfected roots of the two sugar beet cultivars (UnR vs UnS) at 4 dai. At 10 dai, the comparison of uninfected roots of the two cultivars (UnR vs UnS) led to the identification of 337 DEGs. Among them several genes, including examples encoding caffeoyl-CoA O-methyltransferase, dirigent proteins, chalcone synthases, tricetin 3′,4′,5′-O-trimethyltransferase, citrate synthase and endopeptidase inhibitors, showed higher expression in uninfected resistant plants compared to the susceptible plants at both time points (Additional file 5: Table S5). Using a similar comparison between infected plants of both cultivars (IR vs IS), 295 DEGs were found at 4 dai and 247 at 10 dai. Among these genes, a methyltransferase, phosphoinositide phosphatase SAC6, genes related to the phenylpropanoid pathway (such as caffeoyl-CoA O-methyltransferase and anthocyanin 3′-O-beta-glucosyltransferase), genes involved in signaling (including calcium-binding proteins, protein phosphatases 2C-type, protein phosphatase methylesterase and lactosylceramide 4-alpha-galactosyltransferase), F-box proteins, aquaporins, papain-like cysteine protease and some unknown genes that were highly induced in IR vs IS at 4, 10 or both time points (Additional file 5: Table S5).

To identify candidate genes related to the resistance, we compared the BCN-induced responses in both cultivars and looked for stronger or exclusive induction in resistant cultivar Nemakill. An aquaporin, bisphosphate carboxylase small chain, F-box protein, receptor-like protein kinase, trypsin inhibitor, galactono-1,4-lactone dehydrogenase, CASP-like protein, chitinase, and 16 unknown genes were highly induced in IR vs IS at 4 dai (6.89 ≥ log2FC ≥ 2.0, Additional file 6: Table S6). Three putative disease resistance genes, three HSPs, a nucleoredoxin, ET-responsive transcription factor, multiprotein bridging factor 1, and one unknown gene were strongly induced in IR vs IS at 10 dai (6.02 ≥ log2FC ≥ 2.19, Additional file 6: Table S6). CYSTM domain-containing proteins, 14–3-3 protein, an ABC transporter, UDP-glucose flavonoid 3-O-glucosyltransferase and some unknown genes were also only induced in the infected resistant roots comparing to their controls. These genes could be responsible for the previously confirmed incompatible response of cultivar Nemakill [29, 31, 37, 38].

### Validation of the transcriptome data

Quantitative real time-PCR (qRT-PCR) was performed to validate the mRNA-seq data. From the 10 initially selected genes, specific amplification was successful for six transcripts. The results of data analysis confirmed consistent expression patterns between the qRT-PCR and mRNA-seq data (Table 2).

**Table 2:** Expression profiles of six selected genes in the *Heterodera schachtii*-infected and uninfected sugar beet roots at 4 dai, as determined by qRT-PCR and mRNA-seq

Gene ID (RefBeet1.2)	Gene description	log2 fold change							
		UnR vs UnS		IR vs IS		IR vs UnR		IS vs UnS	
		qRT-PCR	mRNA-seq	qRT-PCR	mRNA-seq	qRT-PCR	mRNA-seq	qRT-PCR	mRNA-seq
BVRB_003160	Polymerase	0.57	0.06	3.11	3.14	1.15	0.91	−1.39	−0.65
BVRB_004820	Defense response	− 1.57	− 0.77	− 1.60	−1.61	−0.31	−0.77	−0.28	0.05
BVRB_3g067160	Binding to nucleic acid	−1.51	−2.21	−0.87	−1.55	0.92	0.03	4.23	3.70
BVRB_3g070680	Unknown	−1.97	−0.83	3.68	2.16	1.12	1.03	1.54	0.41
BVRB_4g093270	Unknown	1.70	0.98	−4.04	−2.67	−0.73	−0.06	4.98	1.86
BVRB_9g225900	Unknown	−0.55	0.40	8.20	3.34	0.43	0.07	−8.38	−5.23

*dai* days after infection, *UnR* uninfected resistant roots, *UnS* uninfected susceptible roots, *IR* infected resistant roots, *IS* infected susceptible roots

### Detection of *H. schachtii* transcripts in infected roots of sugar beets

Non-sugar beet mapped reads of the infected susceptible and resistant roots were further analyzed as explained in details in the Materials and Methods section. In total, 1707 transcripts of *H. schachtii* were identified from the infected samples. At early stage (4 dai), 342 and 469 genes were identified in the susceptible and in the resistant samples, respectively. At this time point, 211 nematode genes were common between both cultivars (Additional file 7: Table S7). At late stage (10 dai), 1321 and 901 nematode transcripts were identified in the susceptible and resistant samples, respectively. There were 323 common nematode genes between the cultivars (Additional file 7: Table S7).

Several nematode housekeeping genes including actin, tubulin, ubiquitin, ribosomal proteins, initiation and elongation factors and heat shock proteins were expressed in all infected sugar beet roots of both cultivars. Also, some genes involved in transport and lipid metabolism were identified. Among the transcripts, some genes encoding cell-wall degrading enzymes (cell wall-associated hydrolases and polygalacturonases) and peptidases (aspartic, metallo and serine) were detected (Additional file 7: Table S7).

Regarding to parasitism, genes encoding antioxidants such as thioredoxin-related transmembrane protein (in all treatments), superoxide dismutase (IS-10 dai and IR-10 dai), glutathione S-transferase (IS-10 dai and IR-10 dai), and cytochrome C-peroxidases were detected. In addition, several known or putative effector genes were found, including transcripts encoding cathepsin (one transcript in IS-4 dai, two transcripts in IR-4 dai, three transcripts in IS-10 dai and IR-10 dai), ubiquitin extension protein (IS-4 dai, IR-4 dai, IS-10 dai), 14–3-3 protein (one transcript in IS-4 dai, IR-4 dai and IR-10 dai and two transcripts in IS-10 dai), calreticulin (two transcripts in IR-4 dai, IS-10 dai and IR-10 dai), C-type lectins (two transcripts in IS-10 dai and IR-10 dai), transthyretin-like protein (five transcripts in IS10, three transcripts in IR10), esophageal gland cell secretory proteins (six transcripts in IS-10 dai and three transcripts in IR-10 dai), and uncharacterized putative effector proteins (two transcripts in IR-4 dai and IR-10 dai, three transcripts in IS-10 dai). A gene encoding an autophagy-related protein and one encoding a cold-shock-like protein were only detected in the resistant roots at both time points, suggesting that the nematodes are under stress in the incompatible interaction. Also, a gene encoding Bax inhibitor 1 protein, a cell death suppressor, was only detected in nematode transcripts of the infected resistant roots at 10 dai.

## Discussion

The global need for sugar, as one of the most important components of foods and drinks, is increasing rapidly. Sugar beet accounts for almost all sugar production in Europe and for over a quarter (30%) of the total world production. Among the factors that reduce the sugar beet yield, *H. schachtii* (BCN) is known as a major limiting factor, yet little is known about the interaction between sugar beet and BCN at the molecular level [36]. To increase our knowledge, transcriptome analyses were carried out using next generation sequencing (NGS) technology to investigate the compatible and incompatible sugar beet reaction to infection with *H. schachtii* and as a result in total about 443 million bp raw reads were acquired. We identified a large number of genes related to cell wall architecture, metabolism, nutrition, signal transduction, stress, defense responses and phytohormones, for which the expression was significantly altered upon BCN infection. Genes that are only up-regulated in the incompatible interaction but rather unaffected or even suppressed in the compatible interaction could potentially be related to the resistance response of sugar beet against BCN.

For example, three genes encoding CYSTM domain-containing proteins were up-regulated in the resistant roots of sugar beet upon BCN infection. It has been suggested that CYSTM proteins are part of a cellular protective mechanism against stress in eukaryotes, including humans [39]. The *PCC1* gene in Arabidopsis, belonging to this group, encodes an 81-amino acid protein, with a cysteine-rich domain that is involved in development and defense response to stresses including pathogens [40]. So far, the role of these genes in plant-nematode interactions has not been reported.

A gene encoding chitinase, was down-regulated early in the BCN-infected susceptible plants, while significantly up-regulated in the infected resistant plants. Alteration of chitinase gene expression in different plants as a defense mechanism against nematodes has been reported [18, 41]. Guimaraes et al. [42] suggested a role of *AsCHI2* in the defence response of *Arachis stenosperma* to *Meloidogyne arenaria*.

In addition, a gene encoding SKIP23, an F-box protein containing a domain of unknown function (DUF295), was highly induced in the infected resistant plants compared to the susceptible plants at 4 dai. F-box proteins are components of SCF (Skp I, Cullin, and an F-box protein) ubiquitin-ligase (E3) complexes, which mediate ubiquitination and subsequent proteasomal degradation of target proteins. These proteins play diverse roles in different processes such as plant development [43–45], circadian clock regulation [46, 47], hormone perception and signaling [48–53], response to abiotic stresses [54–57] and plant-pathogen interactions [58–61]. The protein SKIP23 was found to interact with ASK1, a component of, for example, the strigolactone SCF receptor complex, [62, 63] and also with 14–3-3 proteins in Arabidopsis [64]. Interestingly, a nematode-derived transcript encoding a 14–3-3 protein was accumulating in the infected resistant roots compared to the controls. The role of the F-box protein SKIP23 in the resistance of sugar beet cultivar Nemakill seems likely although more investigations are needed to elucidate the exact role of this gene. On the other hand, a similar F-box protein with DUF295 domain (Ascorbic acid Mannose Pathway Regulator 1, AMR1) has been shown to be involved in modulating the expression of several genes in the ascorbate biosynthesis pathway in Arabidopsis [65]. Ascorbate is an important natural compound with high antioxidant activity. The main biosynthetic pathway of ascorbate in plants is the “Smirnoff-Wheeler” pathway [66], in which the last step is the oxidation of L-galactono-1,4-lactone to L-ascorbate by the enzyme *L-galactono-1,4-lactone dehydrogenase* (*GLDH*) in the mitochondria [67]. The here-observed suppression of the *GLDH* gene during the compatible interaction, but significant induction during an incompatible interaction at 4 dai confirms a potential role of ascorbate in resistance of sugar beet to *H. schachtii*. Similarly, [68] have shown a significant accumulation of ascorbic acid in roots of root knot nematode-resistant tomato cultivars upon nematode infection, but not in susceptible plants.

Suppression of some genes involved in the phenylpropanoid pathway was observed in the susceptible cultivar, while several phenylpropanoid related genes were induced in the resistant cultivar, such as dirigent proteins, chalcone synthases, caffeoyl-CoA O-methyltransferase, anthocyanin 3′-O-beta-glucosyltransferase and UDP-glucose flavonoid 3-O-glucosyltransferase. This indicates the importance of this pathway in resistance of sugar beet against BCN. Phenylpropanoids are a large class of secondary metabolites including SA, lignin, flavonoids, coumarins, lignans etc. [69]. Suppression of genes in this pathway has been previously observed in compatible plant-RKN interactions [18, 70], while induction was reported in compatible plant-CN or plant-migratory nematode interactions [18, 71].

Induction of a gene encoding a casparian strip membrane domain protein (CASP)-like protein, belonging to “uncharacterized protein family UPF0497” was detected in the infected resistant roots compared to the uninfected resistant plants and to the susceptible plants at 4 dai. CASPs mediate Casparian strip formation, composed of a lignin polymer and acting as para-cellular barrier for selective nutrient uptake and stress resistance, also against nematodes [72]. It also plays a role in the activation of hormone signaling pathways [73–76].

Among the genes involved in hormone pathways, genes related to JA biosynthesis and responses such as lipoxygenases and bHLH TFs were up-regulated in the susceptible roots at early stage while their expression was not induced at later stage. Similarly, Kammerhofer et al. [17] reported up-regulation of genes related to JA biosynthesis early upon nematode inoculation. This induction could be related to a damage response caused by intracellular nematode penetration, or could be related to a plant defense response to nematode presence. Our experiments showed that upon foliar treatment of the susceptible sugar beets with MeJA, infection rates of J2s (at 4 dai) and the number of females (at 21 dai) were significantly lower compared to untreated plants. These results reveal the positive role of JA in systemic defense of sugar beet against *H. schachtii*.

In our transcriptome data, genes belonging to the ET pathway were generally suppressed in the susceptible plants, while ET-responsive TF were induced in the infected resistant roots at 10 dai. To investigate the role of ET in the defense response of sugar beet against BCN, we applied Eth on shoots of susceptible sugar beet plants and our results showed lower J2s infection and female development rates on Eth-treated plants compared to controls, although in one experiment the effect on female development was not significant. In contrast to our results, a higher infection rate at 24 hai and no significant differences in number of penetrating BCN J2s and female counts in Eth-treated Arabidopsis roots has been reported [17]. Indeed, contradictory roles of ET in nematode attraction, feeding site formation and development, and plant defense have been reported [17, 20, 24, 77–83], probably because of its pleiotropic role in development and defense [84]. Taken together, it seems likely that ET is playing a role in defense of sugar beet against BCN but further investigations are needed to reveal the exact role of ET at different stages of nematode and feeding site development and plant defense.

Regarding to the presence of the *Hs1*^*pro-1*^ gene in the resistant cultivar, the expression level of this gene was assessed and our analysis showed that the gene was highly expressed under both infected and non-infected conditions in the resistant cultivar. In addition, three other putative disease resistance genes, two genes with LRR and the other one with LRR and NBS-ARC domains, were significantly induced in the BCN-infected resistant roots compared to the uninfected resistant or infected susceptible roots at 10 dai. These data reveal a potential activation of an R-gene complex in the Nemakill cultivar upon BCN infection.

## Conclusions

This study is the first transcriptome analysis of sugar beet in compatible and incompatible interactions with *H. schachtii* and increases our knowledge of the molecular mechanisms underlying BCN resistance in sugar beet. A large number of DEGs, including many important genes playing potential roles in susceptibility or resistance of sugar beet against BCN were identified. In addition, several genes encoding nematode effectors were identified and some of them were only detected in the resistant roots, suggesting a potential role as *avirulence *(*avr*) protein that needs to be further elucidated. The effective role of application of jasmonate and ethylene in enhancing the basal defense response of sugar beet against BCN was showed. The results of this research extend our knowledge about plant-nematode interactions and can be used for breeding programs targeting BCN resistance in sugar beet.

## Methods

### Plant material and nematode infection

Seeds of a susceptible sugar beet line (7112*SB36, Sugar Beet Seed Institute (SBSI), Iran) and a resistant cultivar (Nemakill, Syngenta) were used in this study. Presence of the *Hs1*^*pro-1*^ gene was confirmed in Nemakill by PCR amplification and Sanger sequencing (genbank accession number MT845291). The susceptible cultivar does not contain this gene. Sugar beet seeds were germinated on sterile soil for 3 days at 28 °C. The seedlings were transferred to SAP substrate (Sand Absorbent Polymer [85];) and were kept in a growth chamber at 25 ± 2 °C, with 16 h light/8 h darkness. A pure population of *H. schachtii* that originated from one cyst was multiplied on a susceptible sugar beet cultivar (Jolgeh, SBSI, Iran) in sterile potting soil under the same conditions. The J2s were harvested from cysts that were soaked in 3 mM ZnCl_2_ to stimulate hatching. Fifteen-day-old roots of the sugar beet plants were inoculated with 300 fresh J2s of *H. schachtii*. Control plants were mock-inoculated with water. Whole root tissue of infected and control plants was collected at two time points, 4 (early stage) and 10 (late stage) dai, were then washed in water, immediately frozen in liquid nitrogen and stored at − 80 °C until RNA extraction. For each time point, two independent biological replicates were collected and each replicate consisted of a pool of six individual plants.

### RNA isolation, library preparation, and sequencing

Total RNA was extracted from whole roots of infected and uninfected plants at 4 and 10 dai with the RNeasy Plant Mini Kit (Qiagen) according to the manufacturer’s protocol, with an additional sonication step for 30 s after addition of buffer RLT. The quantity and quality of each RNA sample was evaluated using a NanoDrop 2000c (Thermo Fisher Scientific, Belgium). For each sample, 2 μg of total RNA was used for library preparation using QuantSeq 3′ mRNA-Seq Library Prep Kit (Lexogen) as following: an oligodT primer containing an Illumina-compatible linker sequence at its 5′ end was hybridized to the RNA and reverse transcription was performed. After first strand synthesis, the RNA was removed and second strand synthesis was initiated by random priming and a DNA polymerase. The random primer also contained an Illumina-compatible linker sequence at its 5′ end. The second strand synthesis was followed by a magnetic bead-based purification step. Amplification of libraries was performed, and addition of barcodes was executed during the PCR amplification step. In the final step, the double-stranded libraries were purified by magnetic beads to remove all reaction components. Quality of the libraries was confirmed using an Agilent Bioanalyzer 2100. After cluster generation, 16 libraries were sequenced on a NextSeq 500 Illumina sequencing platform to produce single-end 76 bp reads. The samples were multiplexed to minimize lane effects. Library construction and sequencing were carried out by the NXTGNT sequencing center (Ghent University, Belgium).

### mRNA-Seq data analysis

**Fig. 5:**
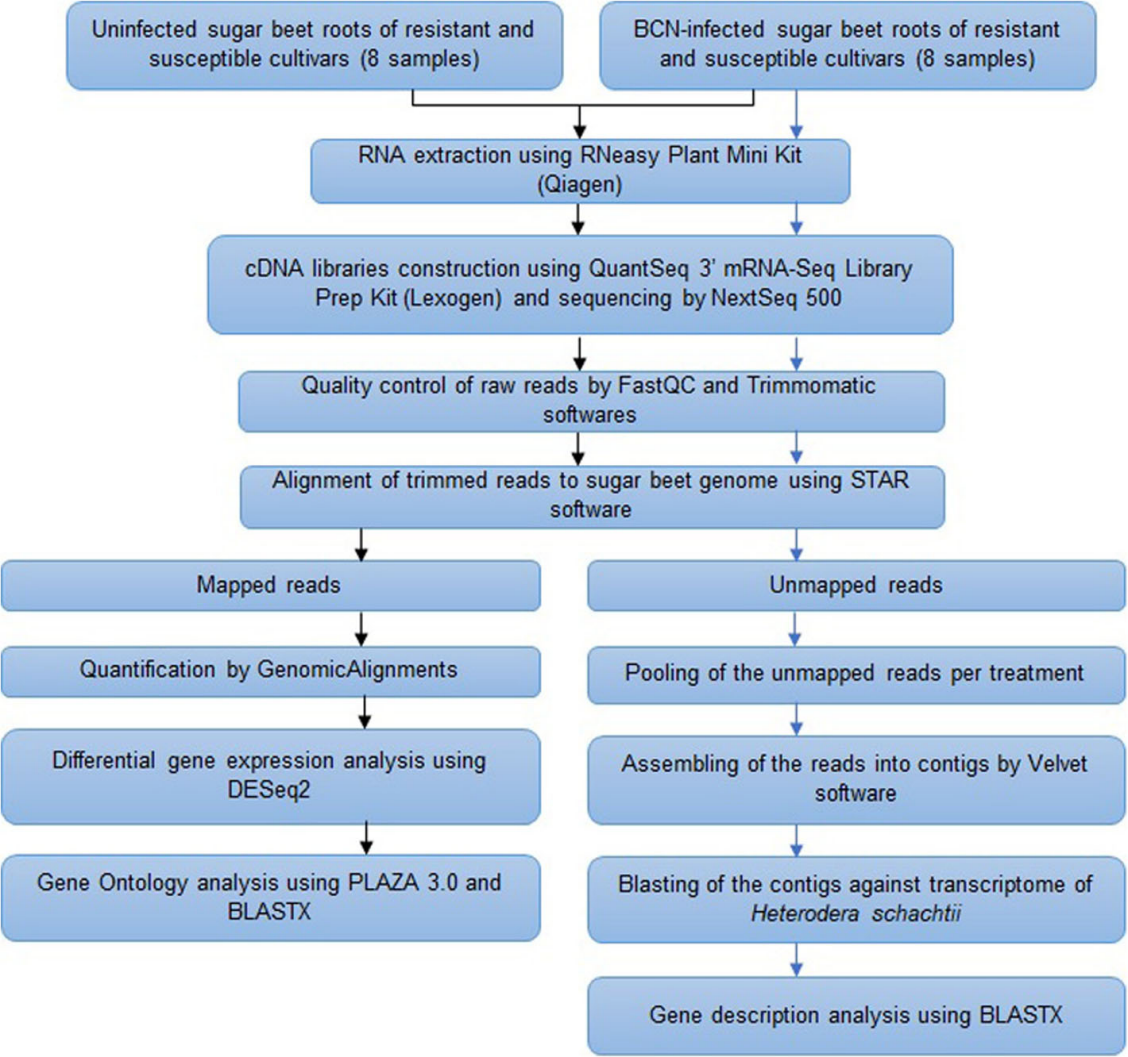
Overview of the mRNA-seq analysis pipeline

A summary of the data-analytical pipeline is shown in Fig. 5. For each sample, sequencing data quality was assessed by FastQC [86]. Trimmomatic [87], with a 5-base sliding window, was used to improve data quality: Bases with a phred score lower than 20 were trimmed, and reads shorter than 40 nt were removed. The trimmed reads from each sample were aligned to the *Beta vulgaris subsp. vulgaris* reference genome (Refbeet1.1, [1]) using the STAR software [88]. Reads that did not map on the sugar beet genome, were kept aside to identify nematode transcripts (see ‘Detection of nematode transcripts in infected root samples’). Transcriptome size was calculated as the sum of the lengths of all primary transcripts.

The number of trimmed reads mapped to each annotated gene per condition was counted using the summarizeOverlaps functions of the GenomicAlignments R package [89]. A gene was considered to be expressed if it had a raw count value higher than 1 in both replicates of each condition. The reads were normalized and differentially expressed genes (DEGs) were identified using the R-package ‘DESeq2’ [90]. In this software, the counts are divided by sample-specific size factors determined by median ratio of gene counts relative to geometric mean per gene. Empirical null modelling was performed using the fdrtool package (version 1.2.15) using the Wald statistic from DESeq2 as input [91]. Adjusted *P*-values for each estimate of False Discovery Rates (FDRs) were calculated using the Benjamin-Hochberg algorithm. Transcripts were considered to be significant DEGs when the adjusted *P*-value was < 0.05. For each time point, the expression level of each transcript in the infected samples was compared to the controls, moderated log_2_-transformed fold-changes (FC) values were further used throughout this study.

### Gene ontology analysis

Gene Ontology (GO) analysis was performed using PLAZA 3.0 (https://bioinformatics.psb.ugent.be/plaza/versions/plaza_v3_dicots/). PLAZA contains information about structural and functional annotation, gene families, protein domains, etc. in plants [92]. Using the PLAZA 3.0 tool, differentially expressed transcripts were assigned to GO categories and functionally annotated. Genes without annotation in PLAZA 3.0 were BLASTX-ed against the non-redundant protein sequences using NCBI online blast service (E-value <1e-5).

### qRT-PCR validation

Based on the mRNA-seq results, 10 genes were selected for validation. Gene-specific primers were designed using Primer3 (http://bioinfo.ut.ee/primer3-0.4.0/) and ApE v2.0.49.10 softwares. Glyceraldehyde 3-phosphate dehydrogenase, *BvGAPDH*, was used as reference gene [93, 94]. For qRT-PCR, independent samples of the susceptible and resistant plants at 4 dai were harvested for RNA-extraction. All reactions were done with two biological and three technical replicates. A total of 2 μg of each RNA sample was treated with 2 U of DNaseI enzyme (Thermofisher). The cDNA synthesis was performed using 200 U of Tetro Reverse Transcriptase enzyme and Oligo (dT)_18_ primer (Tetro cDNA Synthesis kit, Bioline, Germany) according to the manufacturer’s protocol. Quantitative real-time PCR was performed using SensiMix™ SYBR NO-ROX (Bioline, Germany) on a CFX connect real-time PCR machine (Biorad, USA) as following: 10 min of initial denaturation at 95 °C and 40 amplification cycles (25 s at 95 °C, 25 s at 58 °C and 20 s at 72 °C). After the last step, specificity was tested using a melting curve by gradually increasing the temperature to 95 °C. Data were analyzed using Rest 2009 [93]. The expression level of all genes was normalized using the internal control *BvGAPDH* and the relative expression level of target genes was calculated using 2^-ΔΔCt^ method [94]. All primers used in this study are listed in Additional file 8: Table S8.

### Detection of nematode transcripts in infected root samples

The unmapped reads of replicates of each cultivar at each time point were pooled and assembled into contigs using the Velvet software (version 1.2.10 [95];). The contigs were blasted against the transcriptome of *H. schachtii* [96] and those contigs with a bit score higher than 50 were considered as transcripts of *H. schachtii*. BLASTX was performed against the non-redundant protein sequences using NCBI online blast service (E-value <1e-5).

### Chemical treatments and statistical analyses

The susceptible sugar beet line (7112*SB36) was used. Seedlings were transferred to sterile soil in polyvinyl-chloride (PVC) tubes and further grown at the same conditions as described above. Solutions of methyl jasmonate (MeJA, 100 μM, Sigma-Aldrich), or ET-generator ethephon (Eth, 500 μM, Sigma-Aldrich) were prepared in distilled water containing 0.02% (v/v) Tween 20 as surfactans. Leaves of two-week-old sugar beet plants were sprayed with vaporizers until run off with a fine mist of either compound. For control plants, distilled water containing 0.02% (v/v) Tween 20 was applied. One day after chemical treatments, roots of sugar beet plants were inoculated with 300 fresh J2s, as described in previous steps. The infection level of the plants was evaluated at 4 and 21 dai by counting the number of J2s and females, respectively. To visualize the J2s, roots were stained with acid fuchsin at 4 dai [22]. Ten plants per treatment were included in each experiment and all experiments were independently repeated three times.

Statistical analyses were done using SPSS Statistics V22.0 software. After confirming normality and homoscedasticity of the data, one-way ANOVA and Duncan‘s multiple range test were applied to test for significant differences between the treatments (*P* < 0.05).

## Supplementary information

**Additional file 1 Table S1.** Significant differentially expressed genes (DEGs) of sugar beet in the compatible sugar beet-beet cyst nematode (BCN) interaction.

**Additional file 2 Table S2.** Significant sugar beet DEGs related to cell wall architecture, metabolism, developmental process, transport, defense response, hormone metabolism and transcription factors in the compatible sugar beet- BCN interaction.

**Additional file 3 Table S3.** Significant sugar beet DEGs in the incompatible sugar beet- BCN interaction.

**Additional file 4 Table S4.** Significant differentially expressed sugar beet genes related to cell wall architecture, transport, defense response, hormone metabolism and transcription factors in the incompatible sugar beet- BCN interaction.

**Additional file 5 Table S5.** Significant DEGs in comparison of resistant and susceptible sugar beet cultivars in uninfected and BCN-infected conditions.

**Additional file 6 Table S6.** Sugar beet genes that show a differential response to BCN infection when comparing the resistant and susceptible cultivar. Statistical significance was evaluated in DESeq2 by evaluating significant interaction between infections status and sugar beet variety.

**Additional file 7 Table S7.** Transcripts derived from beet cyst nematode (BCN) genes detected during the compatible and incompatible interaction with sugar beet.

**Additional file 8 Table S8.** Primer sequences of sugar beet genes used for qRT-PCR expression analysis.

## Data Availability

The partial sequence of the *Hs1*^*pro-1*^ gene from sugar beet cultivar Nemakill has been deposited at the GenBank database with accession number MT845291. The mRNA datasets generated during the current study are available at the NCBI Gene Expression Omnibus (GEO) under the accession number GSE135555. The *Beta vulgaris subsp. vulgaris* reference genome and the transcriptome of *H. schachtii* were obtained from the http://bvseq.boku.ac.at/Genome/Download/RefBeet-1.1/ and https://www.ncbi.nlm.nih.gov/sra/?term=SRX381021, respectively.

## Abbreviations

BCN: Beet cyst nematode
dai: Days after inoculation
DEG: Differentially expressed gene
ET: Ethylene
Eth: Ethephon
IS: Infected susceptible roots
IR: Infected resistant roots
JA: Jasmonic acid
MeJA: Methyl jasmonate
qRT-PCR: Quantitative real time-PCR
TF: Transcription factor
UnR: Uninfected resistant roots
UnS: Uninfected susceptible roots

## References

[1] Dohm JC, Minoche AE, Holtgrawe D, Capella-Gutierrez S, Zakrzewski F, Tafer H, Rupp O, Sorensen TR, Stracke R, Reinhardt R (2014). The genome of the recently domesticated crop plant sugar beet (*Beta vulgaris*). Nature..

[2] Food and Agriculture Organization of the United Nations. FAOSTAT Statistical Database. [Rome] [http://www.fao.org/faostat/en ].

[3] Abad P, Gouzy J, Aury JM, Castagnone-Sereno P, Danchin EG, Deleury E, Perfus-Barbeoch L, Anthouard V, Artiguenave F, Blok VC (2008). Genome sequence of the metazoan plant-parasitic nematode *Meloidogyne incognita*. Nat Biotechnol.

[4] Schmidt A (1871). About the beet nematode. Zollverein..

[5] Schacht H (1859). Über einige Feinde der Rübenfelder. Zeitschrift Vereines Rübenzucker-Industrie Zollverein.

[6] Biancardi E, McGrath JM, Panella LW, Lewellen RT, Stevanato P. Sugar beet. In: Root and tuber crops. New York: Springer-Verlag; 2010. p. 173–219.

[7] Franklin MT. *Heterodera schachtii*. In: C.I.H. Descriptions of Plant Parasitic Nematodes. Wallingford CAB international, n.d. St. Albans: Commonwealth Institute of Helminthology; 1972. p. 4.

[8] Wyss U, Grundler FMW (1992). Feeding behavior of sedentary plant parasitic nematodes. Neth J Plant Pathol.

[9] Hussey RS, Grundler FM. Nematode parasitism of plants. In: Perry RN, Wright DJ, editors. The Physiology and Biochemistry of Free-living and Plant-parasitic nematodes. 1st ed. Wallingford: CABI publishing; 1998. p. 213–43.

[10] Ali S, Magne M, Chen S, Cote O, Stare BG, Obradovic N, Jamshaid L, Wang X, Belair G, Moffett P (2015). Analysis of putative apoplastic effectors from the nematode, *Globodera rostochiensis*, and identification of an expansin-like protein that can induce and suppress host defenses. PLoS One.

[11] Bekal S, Niblack TL, Lambert KN (2003). A chorismate mutase from the soybean cyst nematode *Heterodera glycines* shows polymorphisms that correlate with virulence. Mol Plant-Microbe Interact.

[12] Jaouannet M, Magliano M, Arguel MJ, Gourgues M, Evangelisti E, Abad P, Rosso MN (2013). The root-knot nematode calreticulin mi-CRT is a key effector in plant defense suppression. Mol Plant-Microbe Interact.

[13] Mitreva-Dautova M, Roze E, Overmars H, de Graaff L, Schots A, Helder J, Goverse A, Bakker J, Smant G (2006). A symbiont-independent endo-1,4-beta-xylanase from the plant-parasitic nematode *Meloidogyne incognita*. Mol Plant-Microbe Interact.

[14] Qin L, Kudla U, Roze EH, Goverse A, Popeijus H, Nieuwland J, Overmars H, Jones JT, Schots A, Smant G (2004). Plant degradation: a nematode expansin acting on plants. Nature..

[15] Rosso M-N, Grenier E. Other nematode effectors and evolutionary constraints. In: Genomics and molecular genetics of plant-nematode interactions. The Netherlands: Springer; 2011. p. 287–307.

[16] Ji H, Kyndt T, He W, Vanholme B, Gheysen G (2015). Beta-Aminobutyric acid-induced resistance against root-knot nematodes in Rice is based on increased basal defense. Mol Plant-Microbe Interact.

[17] Kammerhofer N, Radakovic Z, Regis JM, Dobrev P, Vankova R, Grundler FM, Siddique S, Hofmann J, Wieczorek K (2015). Role of stress-related hormones in plant defence during early infection of the cyst nematode *Heterodera schachtii* in Arabidopsis. New Phytol.

[18] Kyndt T, Denil S, Haegeman A, Trooskens G, Bauters L, Van Criekinge W, De Meyer T, Gheysen G (2012). Transcriptional reprogramming by root knot and migratory nematode infection in rice. New Phytol.

[19] Wubben MJ, Jin J, Baum TJ (2008). Cyst nematode parasitism of *Arabidopsis thaliana* is inhibited by salicylic acid (SA) and elicits uncoupled SA-independent pathogenesis-related gene expression in roots. Mol Plant-Microbe Interact.

[20] Wubben MJ, Su H, Rodermel SR, Baum TJ (2001). Susceptibility to the sugar beet cyst nematode is modulated by ethylene signal transduction in *Arabidopsis thaliana*. Mol Pant-Microbe Interact.

[21] Cooper WR, Jia L, Goggin L (2005). Effects of jasmonate-induced defenses on root-knot nematode infection of resistant and susceptible tomato cultivars. J Chem Ecol.

[22] Nahar K, Kyndt T, Nzogela YB, Gheysen G (2012). Abscisic acid interacts antagonistically with classical defense pathways in rice-migratory nematode interaction. New Phytol.

[23] Soriano IR, Asenstorfer RE, Schmidt O, Riley IT (2004). Inducible flavone in oats (Avena sativa) is a novel defense against plant-parasitic nematodes. Phytopathology..

[24] Goverse A, Overmars H, Engelbertink J, Schots A, Bakker J, Helder J (2000). Both induction and morphogenesis of cyst nematode feeding cells are mediated by auxin. Mol Plant-Microbe Interact.

[25] Hermsmeier D, Hart JK, Byzova M, Rodermel SR, Baum TJ (2000). Changes in mRNA abundance within *Heterodera schachtii*-infected roots of *Arabidopsis thaliana*. Mol Plant-Microbe Interact.

[26] Puthoff DP, Nettleton D, Rodermel SR, Baum TJ (2003). Arabidopsis gene expression changes during cyst nematode parasitism revealed by statistical analyses of microarray expression profiles. Plant J.

[27] Szakasits D, Heinen P, Wieczorek K, Hofmann J, Wagner F, Kreil DP, Sykacek P, Grundler FM, Bohlmann H (2009). The transcriptome of syncytia induced by the cyst nematode *Heterodera schachtii* in Arabidopsis roots. Plant J.

[28] Doré C, Varoquaux F (2006). Histoire et amélioration de cinquante plantes cultivées.

[29] Pylypenko L, Kalatur K (2015). Breeding and usage of sugar beet cultivars and hybrids resistant to sugar beet nematode Heterodera schachtii. Agricult Sci Pract.

[30] Pylypenko L, Kalatur K, Hallmann J (2016). Sugar beet nematode Heterodera schachtii distribution and harmfulness in Ukraine. Agricult Sci Pract.

[31] Schlang J (1999). Keine Chance für Nematoden. DLZ-Agrarmagazin..

[32] Cai D, Kleine M, Kifle S, Harloff H-J, Sandal NN, Marcker KA, Klein-Lankhorst RM, Salentijn EM, Lange W, Stiekema WJ (1997). Positional cloning of a gene for nematode resistance in sugar beet. Science..

[33] Capistrano GGG (2010). A candidate sequence for the nematode resistance gene Hs1–2 in sugar beet.

[34] Jäger S (2013). Hybrid assembly of whole genome shotgun sequences of two sugar beet (*Beta vulgaris* L.) translocation lines carrying the beet cyst nematode resistance gene Hs1–2 and functional analysis of candidate genes.

[35] Yu MH, Steele AE (1981). Host-parasite interaction of resistant Sugarbeet and Heterodera schachtii. J Nematol.

[36] Samuelian S, Kleine M, Ruyter-Spira CP, Klein-Lankhorst RM, Jung C (2004). Cloning and functional analyses of a gene from sugar beet up-regulated upon cyst nematode infection. Plant Mol Biol.

[37] Doré C, Varoquaux F: Histoire et amélioration de cinquante plantes cultivées. : Quae; 2006.

[38] Ghaemi R, Pourjam E, Safaie N, Mahmoudi SB, Mehrabi R (2018). Evaluation of sugar beet cultivars resistance to beet cyst nematode under in vitro conditions. J Sugar Beet.

[39] Venancio TM, Aravind L (2010). CYSTM, a novel cysteine-rich transmembrane module with a role in stress tolerance across eukaryotes. Bioinformatics..

[40] Mir R, Leon J (2014). Pathogen and circadian controlled 1 (PCC1) protein is anchored to the plasma membrane and interacts with subunit 5 of COP9 signalosome in Arabidopsis. PLoS One.

[41] Li R, Rashotte AM, Singh NK, Lawrence KS, Weaver DB, Locy RD (2015). Transcriptome Analysis of Cotton (*Gossypium hirsutum* L.) Genotypes That Are Susceptible, Resistant, and Hypersensitive to Reniform Nematode (*Rotylenchulus reniformis*). PLoS One.

[42] Guimaraes PM, Guimaraes LA, Morgante CV, Silva OB, Araujo AC, Martins AC, Saraiva MA, Oliveira TN, Togawa RC, Leal-Bertioli SC (2015). Root Transcriptome analysis of wild Peanut reveals candidate genes for nematode resistance. PLoS One.

[43] Durfee T, Roe JL, Sessions RA, Inouye C, Serikawa K, Feldmann KA, Weigel D, Zambryski PC (2003). The F-box-containing protein UFO and AGAMOUS participate in antagonistic pathways governing early petal development in Arabidopsis. Proc Natl Acad Sci U S A.

[44] Gonzalez-Carranza ZH, Rompa U, Peters JL, Bhatt AM, Wagstaff C, Stead AD, Roberts JA (2007). Hawaiian skirt: an F-box gene that regulates organ fusion and growth in Arabidopsis. Plant Physiol.

[45] Samach A, Klenz JE, Kohalmi SE, Risseeuw E, Haughn GW, Crosby WL (1999). The UNUSUAL FLORAL ORGANS gene of Arabidopsis thaliana is an F-box protein required for normal patterning and growth in the floral meristem. Plant J.

[46] Baudry A, Ito S, Song YH, Strait AA, Kiba T, Lu S, Henriques R, Pruneda-Paz JL, Chua NH, Tobin EM (2010). F-box proteins FKF1 and LKP2 act in concert with ZEITLUPE to control Arabidopsis clock progression. Plant Cell.

[47] Imaizumi T, Tran HG, Swartz TE, Briggs WR, Kay SA (2003). FKF1 is essential for photoperiodic-specific light signalling in Arabidopsis. Nature..

[48] Binder BM, Walker JM, Gagne JM, Emborg TJ, Hemmann G, Bleecker AB, Vierstra RD (2007). The Arabidopsis EIN3 binding F-box proteins EBF1 and EBF2 have distinct but overlapping roles in ethylene signaling. Plant Cell.

[49] Dill A, Thomas SG, Hu J, Steber CM, Sun TP (2004). The Arabidopsis F-box protein SLEEPY1 targets gibberellin signaling repressors for gibberellin-induced degradation. Plant Cell.

[50] Kepinski S, Leyser O (2005). The Arabidopsis F-box protein TIR1 is an auxin receptor. Nature..

[51] Koops P, Pelser S, Ignatz M, Klose C, Marrocco-Selden K, Kretsch T (2011). EDL3 is an F-box protein involved in the regulation of abscisic acid signalling in Arabidopsis thaliana. J Exp Bot.

[52] Nelson DC, Scaffidi A, Dun EA, Waters MT, Flematti GR, Dixon KW, Beveridge CA, Ghisalberti EL, Smith SM (2011). F-box protein MAX2 has dual roles in karrikin and strigolactone signaling in Arabidopsis thaliana. Proc Natl Acad Sci U S A.

[53] Thines B, Katsir L, Melotto M, Niu Y, Mandaokar A, Liu G, Nomura K, He SY, Howe GA, Browse J (2007). JAZ repressor proteins are targets of the SCF (COI1) complex during jasmonate signalling. Nature..

[54] Bu Q, Lv T, Shen H, Luong P, Wang J, Wang Z, Huang Z, Xiao L, Engineer C, Kim TH (2014). Regulation of drought tolerance by the F-box protein MAX2 in Arabidopsis. Plant Physiol.

[55] Calderon-Villalobos LI, Nill C, Marrocco K, Kretsch T, Schwechheimer C (2007). The evolutionarily conserved Arabidopsis thaliana F-box protein AtFBP7 is required for efficient translation during temperature stress. Gene..

[56] Maldonado-Calderon MT, Sepulveda-Garcia E, Rocha-Sosa M (2012). Characterization of novel F-box proteins in plants induced by biotic and abiotic stress. Plant Sci.

[57] Zhao Z, Zhang G, Zhou S, Ren Y, Wang W (2017). The improvement of salt tolerance in transgenic tobacco by overexpression of wheat F-box gene TaFBA1. Plant Sci.

[58] Cao Y, Yang Y, Zhang H, Li D, Zheng Z, Song F (2008). Overexpression of a rice defense-related F-box protein gene OsDRF1 in tobacco improves disease resistance through potentiation of defense gene expression. Physiol Plant.

[59] Curtis RH (2013). Pankaj, powers SJ, Napier J, Matthes MC: the Arabidopsis F-box/Kelch-repeat protein At2g44130 is upregulated in giant cells and promotes nematode susceptibility. Mol Plant-Microbe Interact.

[60] Piisila M, Keceli MA, Brader G, Jakobson L, Joesaar I, Sipari N, Kollist H, Palva ET, Kariola T (2015). The F-box protein MAX2 contributes to resistance to bacterial phytopathogens in Arabidopsis thaliana. BMC Plant Biol.

[61] Stefanowicz K, Lannoo N, Zhao Y, Eggermont L, Van Hove J, Al Atalah B, Van Damme EJ (2016). Glycan-binding F-box protein from Arabidopsis thaliana protects plants from Pseudomonas syringae infection. BMC Plant Biol.

[62] Risseeuw EP, Daskalchuk TE, Banks TW, Liu E, Cotelesage J, Hellmann H, Estelle M, Somers DE, Crosby WL (2003). Protein interaction analysis of SCF ubiquitin E3 ligase subunits from Arabidopsis. Plant J.

[63] Yao R, Ming Z, Yan L, Li S, Wang F, Ma S, Yu C, Yang M, Chen L, Chen L (2016). DWARF14 is a non-canonical hormone receptor for strigolactone. Nature..

[64] Hong JP, Adams E, Yanagawa Y, Matsui M, Shin R (2017). AtSKIP18 and AtSKIP31, F-box subunits of the SCF E3 ubiquitin ligase complex, mediate the degradation of 14-3-3 proteins in Arabidopsis. Biochem Biophys Res Commun.

[65] Zhang W, Lorence A, Gruszewski HA, Chevone BI, Nessler CL (2009). AMR1, an Arabidopsis gene that coordinately and negatively regulates the mannose/l-galactose ascorbic acid biosynthetic pathway. Plant Physiol.

[66] Wheeler GL, Jones MA, Smirnoff N (1998). The biosynthetic pathway of vitamin C in higher plants. Nature..

[67] Mapson LW, Isherwood FA, Chen YT (1954). Biological synthesis of L-ascorbic acid: the conversion of L-galactono-gamma-lactone into L-ascorbic acid by plant mitochondria. Biochem J.

[68] Arrigoni O, Zacheo G, Arrigoni-Liso R, Bleve-Zacheo T, Lamberti F (1979). Relationship between ascorbic acid and resistance in tomato plants to Meloidogyne incognita. Phytopathology..

[69] Fraser CM, Chapple C (2011). The phenylpropanoid pathway in Arabidopsis. Arabidopsis Book.

[70] Barcala M, Garcia A, Cabrera J, Casson S, Lindsey K, Favery B, Garcia-Casado G, Solano R, Fenoll C, Escobar C (2010). Early transcriptomic events in microdissected Arabidopsis nematode-induced giant cells. Plant J.

[71] Ithal N, Recknor J, Nettleton D, Hearne L, Maier T, Baum TJ, Mitchum MG (2007). Parallel genome-wide expression profiling of host and pathogen during soybean cyst nematode infection of soybean. Mol Plant-Microbe Interact.

[72] Holbein J, Franke RB, Marhavý P, Fujita S, Górecka M, Sobczak M, Geldner N, Schreiber L, Grundler FMW, Siddique S (2019). Root endodermal barrier system contributes to defence against plant-parasitic cyst and root-knot nematodes. Plant J.

[73] Dinneny JR (2014). A gateway with a guard: how the endodermis regulates growth through hormone signaling. Plant Sci.

[74] Robbins NE, Trontin C, Duan L, Dinneny JR (2014). Beyond the barrier: communication in the root through the endodermis. Plant Physiol.

[75] Roppolo D, De Rybel B, Denervaud Tendon V, Pfister A, Alassimone J, Vermeer JE, Yamazaki M, Stierhof YD, Beeckman T, Geldner N (2011). A novel protein family mediates Casparian strip formation in the endodermis. Nature..

[76] Yang J, Ding C, Xu B, Chen C, Narsai R, Whelan J, Hu Z, Zhang M (2015). A Casparian strip domain-like gene, CASPL, negatively alters growth and cold tolerance. Sci Rep.

[77] Ali MA, Abbas A, Kreil DP, Bohlmann H. Overexpression of the transcription factor RAP2.6 leads to enhanced callose deposition in syncytia and enhanced resistance against the beet cyst nematode *Heterodera schachtii* in Arabidopsis roots. BMC Plant Biol. 2013, 13:47. 10.1186/1471-2229-13-47 PMC3623832 23510309

[78] Fudali SL, Wang C, Williamson VM (2013). Ethylene signaling pathway modulates attractiveness of host roots to the root-knot nematode *Meloidogyne hapla*. Mol Plant-Microbe Interact.

[79] Glazer I, Apelbaum A, Orion D (1985). Effect of inhibitors and stimulators of ethylene production on gall development in *Meloidogyne javanica*-infected tomato roots. J Nematol.

[80] Glazer I, Orion D, Apelbaum A (1983). Interrelationships between ethylene production, gall formation, and root-knot nematode development in tomato plants infected with *Meloidogyne javanica*. J Nematol.

[81] Hu Y, You J, Li C, Williamson VM, Wang C (2017). Ethylene response pathway modulates attractiveness of plant roots to soybean cyst nematode *Heterodera glycines*. Sci Rep.

[82] Marhavý P, Kurenda A, Siddique S, Dénervaud Tendon V, Zhou F, Holbein J, Hasan MS, Grundler FM, Farmer EE, Geldner N. Single-cell damage elicits regional, nematode-restricting ethylene responses in roots. EMBO J. 2019:e100972. 10.15252/embj.2018100972 PMC6518030 31061171

[83] Nahar K, Kyndt T, De Vleesschauwer D, Hofte M, Gheysen G (2011). The jasmonate pathway is a key player in systemically induced defense against root knot nematodes in rice. Plant Physiol.

[84] Kyndt T, Vieira P, Gheysen G, de Almeida-Engler J (2013). Nematode feeding sites: unique organs in plant roots. Planta..

[85] Reversat G, Boyer J, Sannier C, Pando-Bahuon A (1999). Use of a mixture of sand and water-absorbent synthetic polymer as substrate for the xenic culturing of plant-parasitic nematodes in the laboratory. Nematology.

[86] Andrews S. FastQC: a quality control tool for high throughput sequence data. Babraham Bioinform. 2010:175–6.

[87] Bolger AM, Lohse M, Usadel B (2014). Trimmomatic: a flexible trimmer for Illumina sequence data. Bioinformatics..

[88] Dobin A, Davis CA, Schlesinger F, Drenkow J, Zaleski C, Jha S, Batut P, Chaisson M, Gingeras TR (2013). STAR: ultrafast universal RNA-seq aligner. Bioinformatics..

[89] Lawrence M, Huber W, Pages H, Aboyoun P, Carlson M, Gentleman R, Morgan MT, Carey VJ (2013). Software for computing and annotating genomic ranges. PLoS Comput Biol.

[90] Love MI, Huber W, Anders S (2014). Moderated estimation of fold change and dispersion for RNA-seq data with DESeq2. Genome Biol.

[91] Strimmer K (2008). Fdrtool: a versatile R package for estimating local and tail area-based false discovery rates. Bioinformatics..

[92] Proost S, Van Bel M, Vaneechoutte D, Van de Peer Y, Inze D, Mueller-Roeber B, Vandepoele K (2015). PLAZA 3.0: an access point for plant comparative genomics. Nucleic Acids Res.

[93] Pfaffl MW, Horgan GW, Dempfle L (2002). Relative expression software tool (REST) for group-wise comparison and statistical analysis of relative expression results in real-time PCR. Nucleic Acids Res.

[94] Schmittgen TD, Livak KJ (2008). Analyzing real-time PCR data by the comparative C(T) method. Nat Protoc.

[95] Zerbino DR, Birney E (2008). Velvet: algorithms for de novo short read assembly using de Bruijn graphs. Genome Res.

[96] Fosu-Nyarko J, Nicol P, Naz F, Gill R, Jones MG (2016). Analysis of the Transcriptome of the infective stage of the beet cyst nematode, *H schachtii*. PLoS One.

